# Associations between breast density and a panel of single nucleotide polymorphisms linked to breast cancer risk: a cohort study with digital mammography

**DOI:** 10.1186/s12885-015-1159-3

**Published:** 2015-03-18

**Authors:** Brad M Keller, Anne Marie McCarthy, Jinbo Chen, Katrina Armstrong, Emily F Conant, Susan M Domchek, Despina Kontos

**Affiliations:** 1 Department of Radiology, University of Pennsylvania Perelman School of Medicine, 3600 Market St. Ste 360, Philadelphia, PA 19104 USA; 2 Department of Medicine, Massachusetts General Hospital, Boston, MA 02114 USA; 3 Department of Biostatistics and Epidemiology, University of Pennsylvania Perelman School of Medicine, Philadelphia, PA 19104 USA; 4 Abramson Cancer Center, University of Pennsylvania Perelman School of Medicine, Philadelphia, PA 19104 USA

**Keywords:** Breast density, Breast cancer, Genetic risk factors, Single-nucleotide polymorphisms, Race-stratified, Association study

## Abstract

**Background:**

Breast density and single-nucleotide polymorphisms (SNPs) have both been associated with breast cancer risk. To determine the extent to which these two breast cancer risk factors are associated, we investigate the association between a panel of validated SNPs related to breast cancer and quantitative measures of mammographic density in a cohort of Caucasian and African-American women.

**Methods:**

In this IRB-approved, HIPAA-compliant study, we analyzed a screening population of 639 women (250 African American and 389 Caucasian) who were tested with a validated panel assay of 12 SNPs previously associated to breast cancer risk. Each woman underwent digital mammography as part of routine screening and all were interpreted as negative. Both absolute and percent estimates of area and volumetric density were quantified on a per-woman basis using validated software. Associations between the number of risk alleles in each SNP and the density measures were assessed through a race-stratified linear regression analysis, adjusted for age, BMI, and Gail lifetime risk.

**Results:**

The majority of SNPs were not found to be associated with any measure of breast density. SNP *rs3817198* (in LSP1) was significantly associated with both absolute area (p = 0.004) and volumetric (p = 0.019) breast density in Caucasian women. In African-American women, SNPs *rs3803662* (in TNRC9/TOX3) and *rs4973768* (in NEK10) were significantly associated with absolute (p = 0.042) and percent (p = 0.028) volume density respectively.

**Conclusions:**

The majority of SNPs investigated in our study were not found to be significantly associated with breast density, even when accounting for age, BMI, and Gail risk, suggesting that these two different risk factors contain potentially independent information regarding a woman’s risk to develop breast cancer. Additionally, the few statistically significant associations between breast density and SNPs were different for Caucasian versus African American women. Larger prospective studies are warranted to validate our findings and determine potential implications for breast cancer risk assessment.

**Electronic supplementary material:**

The online version of this article (doi:10.1186/s12885-015-1159-3) contains supplementary material, which is available to authorized users.

## Background

Breast cancer is currently the most commonly diagnosed cancer and the second leading cause of cancer death in women in the US [1]. Recently, there has been focus on the personalization of breast cancer screening recommendations [2] based on measurable factors known to influence an individual woman’s risk for breast cancer [3]. Of these, breast density has emerged as one of the strongest risk factors for breast cancer [4-15], which can potentially allow for substantial improvements in breast cancer risk estimation.

Mammographic density, the most broadly used measure of breast density, represents the relative amount of radiographically-opaque fibroglandular tissue versus radiographically-translucent adipose tissue in the breast. Commonly measured via visual assessment either qualitatively using the American College of Radiology Breast Imaging-Reporting and Data System (BI-RADS) density categories [16,17], or quantitatively as percent density (PD%) using semi-automated tools [4,18], it has been shown to lead to improvements in breast cancer risk assessment [19-23]. More recently, fully-automated tools have also been developed [13,24,25] which hold the promise to provide more accurate quantitative estimates of density for breast cancer risk evaluation.

To date, the etiological pathways underlying the increase in breast cancer risk due to the presence of dense tissue are not yet clearly understood [26,27]. Breast density is thought to have a polygenic basis [28,29], and identifying which genes are involved in the formation of the dense tissue could elucidate potential pathways linking breast density and breast cancer formation. Genome-wide association studies have identified multiple low and moderate penetrance breast cancer susceptibility loci in women, commonly referred to as single nucleotide polymorphisms (SNPs), associated with both overall and sub-type specific risk [30] that may be useful in breast cancer risk assessment [31-35]. As such, it would be important to determine whether such genetic risk factors are associated with breast density or whether they are potentially independent predictors of a woman’s risk to develop breast cancer.

In this context, we investigate associations between a panel of validated SNPs related to breast cancer risk and quantitative measures of mammographic density in a race-stratified cohort. Given the increasing interest in identifying which measures of breast density are most related to breast cancer risk [36], we evaluate these associations using both area and volumetric density measures. Ultimately, understanding the relationship between breast density and genetic risk factors for breast cancer could provide further insight into the etiological pathways driving the association between breast density and cancer risk. Furthermore, by exploring these associations we can begin to understand how these risk factors relate to each other and how they could be leveraged jointly in breast cancer risk assessment, should they contain independent information.

## Methods

### Study population

In this University of Pennsylvania Institutional Review Board (IRB) approved, HIPAA compliant study, we retrospectively identified a cohort of women aged 40 years or older from our routine breast screening population who had also been prospectively recruited by a separate, IRB-approved, HIPAA compliant clinical study at our institution investigating the added value of genomic markers in breast cancer risk prediction [37]. For the purposes of our study, informed consent was waived, as this was a retrospective analysis and these women were already consented for research purposes in the original study [37] at the time of their recruitment. Each of these women was imaged as part of their routine screening with a full-field digital mammography (FFDM) system (Selenia Dimensions, Hologic Inc.) under a standard protocol. From a total of 810 women originally recruited, a total of 670 had raw (i.e., “FOR PROCESSING”) digital images available on record for quantitative analysis. All these women were interpreted as negative (BI-RADS 1 or 2 screening outcome), and confirmed with at least 1 year follow-up. Information regarding each woman’s current age, demographic and reproductive history, height, weight and race was collected via self-report. Gail lifetime risk, the probability that a woman will develop invasive or in situ breast cancer in a specified time period, was estimated using the National Cancer Institute’s on-line Breast Cancer Risk Assessment Tool [38]. Specifically, the Gail model uses a woman’s current age, age at menarche, age at first live birth, benign breast disease history and family history as predictor variables. In addition, height and weight information was further used to compute body mass index (BMI), categorized as normal weight (BMI < 25 kg/m^2^), overweight (25 kg/m^2^ ≤ BMI < 30 kg/m^2^) and obese (BMI ≥ 30 kg/m^2^). Race information was categorized as Caucasian, African-American or Other; however, given the relatively small number of women who identify as “Other” (N = 31), only women who identified as either Caucasian (N = 389) or African-American (N = 250) were included in this study.

### Genotyping and SNP selection

For each woman, information regarding the genotype of 12 SNPs were obtained from a commercially available assay based on Illumina Infinium II whole-genome genotyping (deCODE BreastCancer, deCODE genetics, Inc.) [37]. The deCODE SNP assay includes 12 genetic loci, specifically 2q35 (*rs13387042*), MRPS30 (*rs4415084*), FGFR2 (*rs1219648*), TNRC9/TOX3 (*rs3803662*), 8q24 (*rs13281615*), LSP1 (*rs3817198*), MAP3K1 (*rs889312*), NEK10 (*rs4973768*), 1p11 (*rs11249433*), RAD51L1 (*rs999737*), COX11/STXBP4 (*rs6504950*), and CASP8 (*rs1045485*), which have been consistently associated with either overall or subtype specific cancer risk, the risk for metastatic disease or age at diagnosis [39-49]. Details of the 12 SNPs investigated in our study are provided in Table 1.

**Table 1:** **Summary of the 12 SNPS in the genetic panel investigated in this study, and their reported associations to breast cancer**

SNP	Gene	Associations to breast cancer
rs1045485	CASP8	Associated with overall breast cancer risk [39]
rs11249433	1p11	Associated with ER+ breast cancer [45,47]
rs1219648	FGFR2	Associated with overall and ER+ breast cancer risk [43,48]
rs13281615	8q24	Associated with ER+, PR+, and low grade tumors [44]
		Associated with survival after diagnosis [44]
rs13387042	2q35	Associated with ER+ risk [40]
rs3803662	TNRC9/TOX3	Associated with ER+ cancer risk and metastatic disease [40]
		Associated with an earlier age at diagnosis [49]
rs3817198	LSP1	Associated with overall breast cancer risk [41]
rs4415084	MRPS30	Associated with ER+ breast cancer [43]
rs4973768	NEK10	Associated with overall breast cancer risk [46]
rs6504950	COX11/STXBP4	Associated with overall breast cancer risk [46]
rs889312	MAP3K1	Associated with overall and ER- breast cancer risk [41,44]
rs999737	RAD51L1	Associated with overall breast cancer risk [45]

The related bibliographic references for each SNP are included in brackets.

### Breast density assessment

Breast density was measured using fully-automated methods. Area-based absolute and percent mammographic density was assessed on a per-image basis using a previously validated, fully-automated algorithm [24]. Briefly, the software automatically delineates the breast region in a digital mammogram from background air and the pectoral muscle. The breast is then subdivided into regions of similar x-ray attenuation via an unsupervised clustering technique, which are then classified into dense and non-dense regions using a support vector machine classifier. The absolute aggregate area of the regions classified as dense, *D*_*A*_, is divided by the total breast area, *B*_*A*_, to obtain a woman’s breast percent density (PD%) using equation 1:

1 $$ PD\%=\frac{D_A}{B_A} $$

These area density estimates acquired per image were averaged across each individual woman’s left and right mediolateral-oblique (MLO) and craniocaudal (CC) screening images in order to obtain a per-woman estimate of both absolute area of dense tissue and PD% for further analysis.

Absolute fibroglandular breast tissue volume and volumetric percent density were also automatically assessed on a per-image basis using fully-automated, FDA-cleared software (Quantra™ v.2.0, Hologic, Inc.) which is based on the widely validated Highnam and Brady method adapted for digital mammography [50,51]. Briefly, this method quantifies the total amount of breast and fibroglandular tissue present within each image pixel via a model of the image acquisition physics and known anatomical properties of the breast and dense tissue. The sum of the breast tissue volume, *B*_*V*_, and fibroglandular dense tissue volume, *D*_*V*_, are then used to calculate the relative volumetric percent density (VD%) seen mammographically via equation 2:

2 $$ VD\%=\frac{D_V}{B_V} $$

As with the area density measures, the individual volumetric density estimates acquired per-image were averaged to obtain corresponding per-woman estimates of absolute fibroglandular tissue volume and VD%.

### Statistical analysis

Differences in age, BMI, Gail lifetime risk, and breast density distributions between the Caucasian and African-American cohorts were assessed using two-sided t-tests with unequal variances for continuous variables and the Chi-squared test for categorical variables at an α = 0.05 significance level. Pearson’s correlation coefficient was used to assess associations between absolute dense area, absolute dense volume, PD% and VD%. Associations between the four breast density measures and each SNP were then assessed with linear regression, in which we adjusted for age, BMI, and Gail lifetime risk by including them as additional covariates in the regression model to determine the significance of the change in density due to the differences in SNP genotype between women in the presence of these additional explanatory variables.

For all analyses, breast density measures were first log-transformed to approximate a normal distribution as has been done in prior works investigating the genetic basis of breast density [29] as well as the association between breast density and risk [13]. The risk allele frequency of each SNP was coded as an ordinal variable (i.e., 0, 1 or 2). In this way, category 0 represents those women homozygous for the common allele of that particular SNP, category 1 represents heterozygous women and category 2 represents women homozygous for the high risk allele. The age and Gail lifetime risk covariates were treated as continuous variables, while BMI category was treated as an ordinal variable. Missing BMI data was handled via race-stratified, standard multiple imputation [52], which replaces missing values with values based on the posterior probability derived from known values [53] within each racial group. For this study, a total of 25 imputations were used, which is greater than the suggested minimum number of 20 [54]. The regression coefficient, confidence interval, and p-value of each SNP was recorded, using the standard α = 0.05 level threshold for significance. Bonferroni correction [55] was also applied to account for multiple comparisons, yielding a second, more stringent significance level cutoff of p = 0.004 (i.e., α = 0.05 divided by 12, the total number of SNPs). In order to assess potential joint associations to breast density, multivariable regression analysis was also performed considering all SNPs and adjusting for age, BMI, and Gail lifetime risk as additional covariates in the regression model. Lastly, to assess the amount of variation in breast density explained by the combination of SNP, age, BMI and Gail lifetime risk, we also computed and report the coefficient of determination, *R*^2^, for each regression model with a significant association to a breast density measure, using a recently proposed method for datasets with multiple imputation [56]. Lastly, given the strong relationship between BMI and breast density, we performed a complete-data analysis to assess whether the associations found in the imputation analysis are maintained when only analyzing those women with known BMI at a lower statistical power. All statistical analyses were performed with STATA 13.1 (StataCorp, College Station, Texas, USA).

## Results

**Figure 1:**
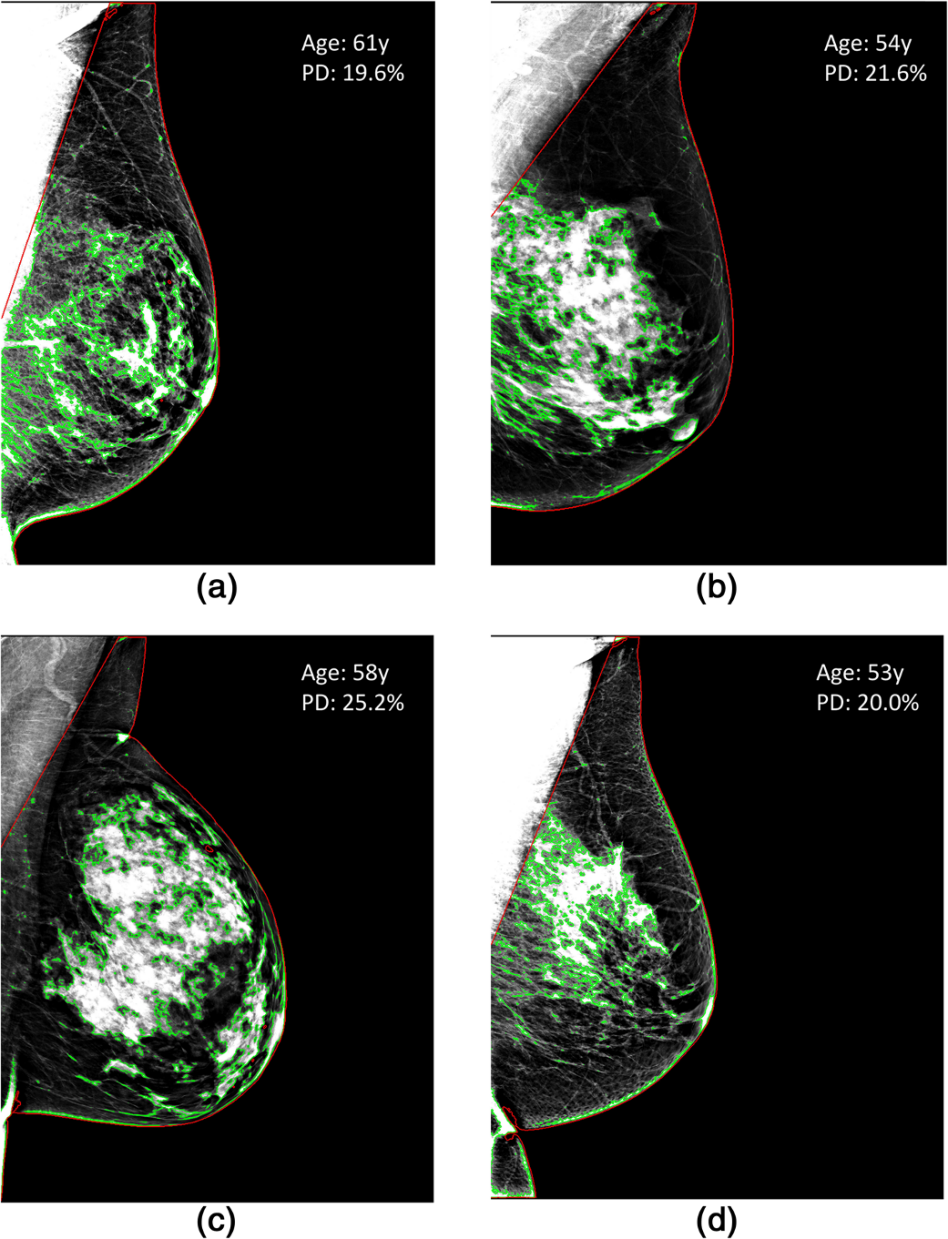
**Area-density segmentations on right, mediolateral-oblique view mammograms for various SNP genotypes.** Four Caucasian women with negative screening exams and different genotypes of *rs3817198* in LSP1 **(a,b)** and *rs1045485* in CASP8 **(c,d)**. Mammograms in the left column **(a,c)** represent women who are homozygous for the common allele for each SNP, while mammograms in the right column **(b,d)** are from women who are homozygous for the risk allele for each SNP. Overall, SNPs, age, Gail life-time risk and BMI were found to only explain a small fraction of the variability in breast density between women. For reference, each woman’s age and overall PD% score are provided as annotations on each image.

Caucasian women were slightly older (p = 0.03), had a lower overall BMI (p < 0.001), and a higher Gail lifetime risk (p < 0.001) than African-American women. When comparing breast density between the two groups, Caucasian women were denser in terms of their percent density both by the area (p < 0.001) and volumetric (p = 0.003) metrics, while African-American women had a greater absolute volume of fibroglandular tissue (p < 0.001). No significant difference was seen between the two groups in terms of absolute area density (p = 0.90). A summary of the demographic and imaging characteristics for the women in our study cohort is shown in Table 2. Statistically significant (p ≤ 0.009) correlations were observed between all the quantitative breast density estimates (Additional file 1: Table S1). Absolute and percent area density had the strongest correlation (r = 0.70, p < 0.001), while absolute and percent volume density had the weakest correlation (r = 0.10, p = 0.009). Figure 1 provides illustrative examples of the dense tissue segmentations in digital mammograms of four representative Caucasian women in our study.

**Table 2:** **Age, BMI and breast density characteristics of the Caucasian and African-American study groups**

	Caucasian	African-American	p-value
**Number of women**	389	250	
**Age (Mean ± SD)**	53.1 y ± 7.1	51.8 y ± 7.6	0.03*
**Gail lifetime risk (Mean ± SD)**	12.1% ± 4.8	8.6% ± 2.8	<0.001*
**Body mass index (BMI)**			<0.001*
*<25 kg/m*^*2*^	197 (50.6%)	36 (14.4%)	
*25-30 kg/m*^*2*^	89 (22.9%)	65 (26%)	
*>30 kg/m*^*2*^	68 (17.5%)	118 (47.2%)	
Missing	35 (9%)	31 (12.4%)	
**Absolute dense area (Mean ± SD)**	30.0 cm^2^ ± 16.6	29.8 cm^2^ ± 16.1	0.90
**Area percent density (Mean ± SD)**	20.1% ± 12.5	14.7% ± 9.7	<0.001*
**Absolute dense volume (Mean ± SD)**	137.7 cm^3^ ± 80.2	215.2 cm^3^ ± 138.0	<0.001*
**Volume percent density (Mean ± SD)**	25.1% ± 10.4	22.7% ± 9.6	0.003*

Pearson χ^2^ test is used to test differences in BMI between the two groups; two-sample t-test with unequal variance is used to test for difference in age, Gail Lifetime Risk and breast density between the groups. * denotes statistical significance at the α = 0.05 level.

**Figure 2:**
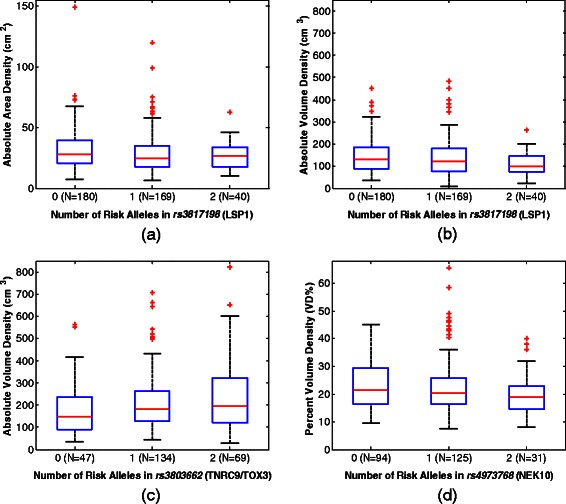
**Box plots illustrating the distribution of breast density measures to significantly associated SNP genotypes. a)** Absolute dense tissue area versus SNP *rs3817198* in Caucasian women; **b)** Absolute volume of dense tissue versus SNP *rs3817198* in Caucasian women; **c)** Absolute volume of dense tissue versus SNP *rs3803662* in African-American women; and **d)** Percent volumetric density (VD%) versus SNP *rs4973768* in African-American women. All box plots provide the median (red-line), interquartile range (blue box), 95% confidence interval (black whiskers) and outliers beyond the 95% confidence interval (red plus-signs).

When assessing associations between area-based density measures and SNPs (Table 3), only one SNP, *rs3817198*, was found to be significantly associated to absolute area density in Caucasian women at the Bonferroni level (p = 0.004, *R*^2^ = 0.07, Figure 2a). This SNP was not found to have a similar association in African-American women (p = 0.175). When assessing associations between volumetric density measures and SNPs, no SNP was found to be significant at the Bonferroni corrected level (Table 4). However *rs3817198* was found to be significantly associated with the absolute volume of dense tissue at the standard significance level in Caucasian women (p = 0.019, *R*^2^ = 0.14, Figure 2b), while it was not significant at either level in African-American women (p = 0.792). In contrast, a different SNP, *rs3803662*, was found to be significantly associated at the standard significance level to absolute volume of dense tissue in African-American women (p = 0.043, *R*^2^ = 0.16, Figure 2c). In addition, SNP *rs4973768* was found to be significantly associated with volumetric percent density at the standard significance level in African-American women (p = 0.028, *R*^2^ = 0.12), but not in Caucasian women (p = 0.680, Figure 2d). Finally, the difference in density score by risk-allele count for those density measures significantly associated with SNPs were confirmed to vary monotonically (Table 5).

**Table 3:** **Regression analysis between number of SNP risk alleles and log-transformed absolute (top) and relative percent (bottom) area density measures in Caucasian (left) and African-American (right) women for each of the 12 SNPs evaluated in this study, after adjusting for age, BMI and Gail lifetime risk**

Absolute area density								
SNP	Caucasian women (N = 389)				African-American women (N = 250)			
	B	p-value	[95% CI]		B	p-value	[95% CI]	
rs1045485	0.002	0.919	−0.041	0.045	−0.014	0.717	−0.088	0.060
rs11249433	−0.014	0.367	−0.045	0.017	−0.016	0.540	−0.065	0.034
rs1219648	−0.014	0.375	−0.046	0.017	0.021	0.300	−0.019	0.061
rs13281615	0.001	0.970	−0.029	0.030	−0.018	0.363	−0.055	0.020
rs13387042	0.014	0.353	−0.015	0.043	0.008	0.679	−0.031	0.048
rs3803662	0.017	0.309	−0.016	0.050	0.024	0.219	−0.014	0.062
***rs3817198***	***−0.047***	***0.004***	***−0.080***	***−0.015***	0.032	0.175	−0.014	0.079
rs4415084	0.021	0.182	−0.010	0.053	−0.003	0.876	−0.039	0.034
rs4973768	−0.005	0.760	−0.035	0.026	−0.035	0.080	−0.074	0.004
rs6504950	0.008	0.650	−0.027	0.043	−0.009	0.633	−0.046	0.028
rs889312	−0.022	0.194	−0.056	0.011	0.003	0.873	−0.037	0.043
rs999737	−0.020	0.280	−0.057	0.016	−0.050	0.262	−0.137	0.037
**Area percent density (PD%)**								
**SNP**	**Caucasian women (N = 389)**				**African-American women (N = 250)**			
	**B**	**p-value**	**[95% CI]**		**B**	**p-value**	**[95% CI]**	
rs1045485	−0.002	0.934	−0.049	0.045	−0.022	0.626	−0.112	0.067
rs11249433	−0.007	0.674	−0.040	0.026	−0.027	0.370	−0.087	0.032
rs1219648	−0.011	0.524	−0.045	0.023	0.008	0.760	−0.041	0.056
rs13281615	−0.014	0.382	−0.046	0.018	0.010	0.659	−0.035	0.056
rs13387042	0.025	0.117	−0.006	0.056	0.004	0.878	−0.045	0.052
rs3803662	0.020	0.269	−0.015	0.055	0.004	0.866	−0.042	0.050
rs3817198	−0.033	0.061	−0.068	0.002	0.046	0.108	−0.010	0.103
rs4415084	0.025	0.149	−0.009	0.059	0.006	0.776	−0.038	0.051
rs4973768	0.015	0.388	−0.018	0.048	−0.029	0.239	−0.077	0.019
rs6504950	0.013	0.504	−0.025	0.050	−0.007	0.768	−0.051	0.037
rs889312	−0.017	0.352	−0.053	0.019	−0.012	0.621	−0.061	0.036
rs999737	−0.015	0.461	−0.054	0.025	−0.038	0.480	−0.142	0.067

The regression coefficient estimate for each individual SNP (B), p-values and the 95% confidence interval of the regression coefficients ([95% CI]) are provided. Significant associations are italicized and bolded.

**Table 4:** **Regression analysis between number of SNP risk alleles and log-transformed absolute (top) and relative percent (bottom) fibroglandular tissue volume in Caucasian (left) and African-American (right) women for each of the 12 SNPs evaluated in this study, after adjusting for age, BMI and Gail lifetime risk**

Absolute volume density								
SNP	Caucasian women (N = 389)				African-American women (N = 250)			
	B	p-value	[95% CI]		B	p-value	[95% CI]	
rs1045485	0.011	0.658	−0.039	0.061	−0.005	0.923	−0.097	0.088
rs11249433	0.015	0.399	−0.020	0.051	0.005	0.879	−0.057	0.066
rs1219648	−0.016	0.398	−0.052	0.021	0.004	0.886	−0.046	0.054
rs13281615	0.007	0.706	−0.027	0.041	−0.043	0.071	−0.090	0.004
rs13387042	0.001	0.941	−0.032	0.035	0.003	0.913	−0.047	0.053
***rs3803662***	0.010	0.599	−0.028	0.048	***0.049***	***0.043***	***0.002***	***0.097***
***rs3817198***	***−0.045***	***0.019***	***−0.082***	***−0.007***	0.008	0.792	−0.051	0.067
rs4415084	0.011	0.567	−0.026	0.047	0.020	0.383	−0.025	0.066
rs4973768	−0.033	0.070	−0.068	0.003	−0.035	0.161	−0.085	0.014
rs6504950	0.008	0.693	−0.032	0.048	0.001	0.964	−0.044	0.047
rs889312	−0.015	0.455	−0.054	0.024	0.027	0.285	−0.023	0.077
rs999737	−0.006	0.797	−0.048	0.037	−0.075	0.176	−0.182	0.033
**Volume percent density (VD%)**								
**SNP**	**Caucasian women (N = 389)**				**African-American women (N = 250)**			
	**B**	**p-value**	**[95% CI]**		**B**	**p-value**	**[95% CI]**	
rs1045485	−0.002	0.907	−0.036	0.032	−0.021	0.492	−0.081	0.039
rs11249433	0.017	0.167	−0.007	0.041	−0.021	0.309	−0.061	0.019
rs1219648	−0.016	0.210	−0.041	0.009	−0.017	0.317	−0.049	0.016
rs13281615	−0.014	0.251	−0.037	0.010	0.011	0.490	−0.020	0.041
rs13387042	0.021	0.072	−0.002	0.044	0.008	0.633	−0.025	0.040
rs3803662	0.008	0.522	−0.017	0.034	0.020	0.200	−0.011	0.051
rs3817198	−0.023	0.076	−0.048	0.002	0.023	0.229	−0.015	0.062
rs4415084	0.017	0.179	−0.008	0.041	0.029	0.057	−0.001	0.058
***rs4973768***	−0.005	0.680	−0.029	0.019	***−0.036***	***0.028***	***−0.068***	***−0.004***
rs6504950	0.005	0.698	−0.022	0.032	0.011	0.483	−0.019	0.040
rs889312	−0.012	0.386	−0.038	0.015	0.001	0.971	−0.032	0.033
rs999737	0.000	0.984	−0.029	0.028	−0.058	0.109	−0.128	0.013

The regression coefficient estimate for each individual SNP (B), p-values and the 95% confidence interval of the regression coefficients ([95% CI]) are provided. Significant associations are italicized and bolded.

**Table 5:** **Mean (μ) and standard deviation (σ) of density measures significantly associated with SNPs by risk allele count**

SNP	Gene	Racial sub-group	Density metric	Density score (μ ± σ) by number of risk alleles		
				0	1	2
*rs3817198*	*LSP1*	Caucasian	Absolute Area Density (cm^2^)	32.0 ± 17.5	28.6 ± 16.6	26.3 ± 11.2
*rs3817198*	*LSP1*	Caucasian	Absolute Volume Density (cm^3^)	145.6 ± 80.5	136.7 ± 84.0	106.7 ± 50.9
*rs3803662*	*TNRC9/TOX3*	African-American	Absolute Volume Density (cm^3^)	187.9 ± 131.6	212.5 ± 129.2	239.0 ± 155.7
*rs4973768*	*NEK10*	African-American	Volume Percent Density (%)	23.3 ± 8.9	23.0 ± 10.4	19.9 ± 8.0

When investigating joint associations between the entire panel of SNPs and each breast density measure through multivariable analysis (Additional files 2, 3, 4, 5: Tables S2-S5), *rs3817198* remained significantly associated to absolute dense area (p = 0.003) and absolute dense volume (p = 0.026) in Caucasian women, and also became significantly associated with area percent density (p = 0.044). SNP *rs3803662* also retained its significance in terms of its association with absolute volume density in African-American women (p = 0.048); while *rs4973768* ceased to be significantly associated with volumetric percent density (p = 0.059). Lastly, complete-data analysis (Additional files 6, 7: Tables S6-S7) showed similar overall trends as the multiple imputation analysis with *rs3817198* remaining significantly associated (p ≤ 0.05) with absolute measures of breast density in Caucasian women, although SNPs *rs3803662* and *rs4973768* only approached significance (p ≤ 0.1) with absolute volume density and volume percent density, respectively, in African-American women, likely due to the decreased sample size in the complete-data analysis leading to a loss of statistical power.

## Discussion

We evaluated potential associations between a panel of validated breast cancer-related SNPs and quantitative measures of volumetric and area-based breast density in a cohort of Caucasian and African-American women. We found that the majority of the SNPs evaluated are not associated with breast density, and that those SNPs that are associated with breast density explain only a small fraction of the total variability in density after accounting for age, BMI and Gail lifetime risk (R^2^: 7%-16%). Specifically, SNP *rs3817198* (in LSP1) was associated with absolute measures of area and volume density in Caucasian women, while in African-American women different SNPs, namely *rs3803662* (in TNRC9/TOX3) and *rs4973768* (in NEK10) were associated with absolute volume of dense tissue and percent volume density, respectively.

Previous studies investigating associations between SNPs and breast density have primarily focused on investigating associations with area-based measures of mammographic density [27,57-62]. These studies have shown a consistent association between breast PD% and SNP *rs3817198* in LSP1 in Caucasian women [27,57-62], as also observed in our study; individual studies have also shown associations between PD% and TNRC9/TOX3*-rs12443621* [57,58] and ZNF365-*rs10995190* [61]. Few studies have also investigated the association between measures of absolute dense area and validated breast cancer risk loci [27,60]. Of these, Vachon et al. [27] observed that a second SNP *rs3803662* in TNRC9/TOX3, a gene also identified in our study, is associated with the absolute amount of dense area. Finally, a recent meta-analysis by Varghese et al. suggested that density has a polygenic basis that likely overlaps at least partially with the genetic basis of breast cancer [28]; although not specifically focusing on which genes and SNPs drive this association, two of the strongest associations were observed with *rs10995190* and *rs10509168*, two SNPs in gene ZNF365 which have also been associated with breast cancer risk [63]. However, these SNPs were not included in the panel assessed in our study, therefore limiting our ability to directly compare with our findings.

While informative, previous studies investigating associations between density and breast cancer susceptibility SNPs have been limited in different aspects: First, most have relied on semi-automated, reader-based [27,57-61] or visual [62] estimates of density, which are known to be sensitive to inter-reader variability [64,65] and may have introduced bias affecting the observed associations. In addition, they have primarily focused on area-based measures of the dense tissue. Given that these measures are a projection estimate of the true volume of fibroglandular tissue, volumetric assessment may provide a more accurate representation of the fibroglandular tissue [66].

In addition, few studies have investigated such associations in African-American women, a population with lower breast cancer incidence but higher mortality rate than Caucasian women [67]. As a result, only some of the SNPs in the panel used in this study have also been validated independently as breast cancer risk factors in African-American women, often with mixed results [48,68-74]. For example, the T allele of rs3803662 (16q12, TOX3), which we have found to be significantly associated with breast density, has also been shown to be significantly associated with a decreased breast cancer risk in African American women but an increased risk in Caucasian women [72], although the finding in African-American women has not been consistently replicated [68-70,75]. In contrast, *rs4973768* in NEK10, which we found to be associated with volumetric percent density in the African-American cohort, has not been found to be associated with breast cancer risk in African-American women [70,72,75]. Regarding the panel as a whole, recent work by our group has found evidence that the 12 SNPs are jointly associated with breast cancer risk in African American women referred for biopsy [76]. Overall, this may suggest that not only may the genetic basis of breast cancer risk vary by race, but the genetic basis of breast density may vary by race as well, potentially allowing for complementary information about breast cancer risk to be ascertained when both genetic and radiographic risk factor information is considered in tandem. Larger studies would be needed to validate this hypothesis.

Although limited by a small sample size, one potentially interesting observation in our study is that the association between SNPs and breast density appears to differ by race, with different SNPs being significant in the two groups even when accounting for age, BMI and Gail lifetime risk. One potential explanation for this observation may be that although there is a large intra-racial variation in density relative to the mean inter-racial differences (Table 2), the genetic basis of density itself may perhaps partially differ in some respects between women of different races, similar to how tumor biology also tends to differ by race [77]. Another possible explanation may be that the total amount of glandular tissue, captured by volumetric density measures, and the spatial distribution of the dense tissue within the breast, captured by area-based measures, could reflect different aspects of the parenchymal pattern originally described by Wolfe [6,14], and thus may represent different aspects of risk related to breast density. Given these open questions, the exploration of potential racial differences in the biology of the different measures of breast density may be worth exploring in future, larger studies.

Although association studies such as ours cannot directly inform on or assess underlying biological processes, they do have value in identifying potential pathways of interest that could be interrogated in subsequent studies through hypothesis generation. For example, LSP1 is thought to play a role in mediating neutrophil activation and chemotaxis, and is expressed in both lymphocytes and endothelium [78], suggesting density may perhaps be, in part, a radiophenotype of genetic risk factors for breast cancer involving tissue vascularization. NEK protein kinases such as NEK10 are thought to play a role in cell cycle regulation [79] and may be related to breast density via factors related to cellular proliferation. Lastly, the protein encoded by TOX3/TNCR9 contains high-mobility-group motif used in altering chromatin structure [80], and thus may be potentially associated with density via some relationship with DNA transcription. Ultimately, a better understanding of the biological pathways could lead to a better understanding of breast oncogenesis as well as the development of better risk assessment tools.

Our study has certain limitations. First, we performed retrospective analysis using data from a single institution. The sample size was also relatively small, which may have limited our power to detect more subtle associations between individual SNPs and density, especially in the context of the race-stratified analysis and the adjustment performed to account for established covariates (i.e., age, BMI, Gail risk). In addition, we only investigated a panel of 12 low-penetrance SNPs associated with breast cancer, while many more risk loci have been recently identified [81]. To fully explore these associations, additional candidate genes related to breast density will also need to be investigated. Furthermore, ancestry informative markers were unfortunately not available for our study to account for population stratification within our Caucasian and African-American sub-cohorts beyond what was already accounted for by a race-stratified analysis. Although not likely to be a major confounder in our study given that genome-wide association studies for breast density have been performed in several populations in which there was little evidence of population stratification [28], they may potentially be of use to account for potential ethnic differences in relatively less-studied African-American or Asian populations and may help explain some of the large intra-racial variation in density relative to the mean inter-racial differences seen in Table 2. Lastly, although breast density is the most common descriptor of the breast parenchyma, genetic variants may also drive other differences in mammographic parenchymal patterns such as texture beyond what can be described by density alone, as previously suggested for the high-penetrance BRCA 1/2 genes [82] and additional SNPs such as rs451632 in the UGT2B gene cluster [83]. Given that parenchymal texture has been shown to be a potentially strong risk factor for breast cancer independent of density [8,84], such texture features may offer another surrogate marker by which the risk conferred by SNPs could manifest radiographically and thus should be considered by future research studies. Overall, larger prospective studies that include parenchymal texture measures as potential radiographic phenotypes of the risk for breast cancer conferred by a more comprehensive panel of known genetic risk factors are warranted to independently validate our findings.

## Conclusion

In conclusion, the majority of the SNPs evaluated in our study were not found to be significantly associated with breast density. Although this may be due to the relatively small sample size of our study, and therefore limited power to detect more subtle associations, our observations suggest that these two risk factors may be capturing potentially independent information regarding a woman’s risk for breast cancer. As such our findings may have implications in the development of future breast cancer risk models by providing evidence that both SNPs and breast density could be considered simultaneously as risk predictors to potentially improve discriminatory capacity. Additionally, our study suggests that the associations between SNPs and breast density appear to differ between Caucasian and African American women. Larger prospective studies are warranted to further validate our findings and determine potential implications for breast cancer risk assessment. Ultimately, understanding the independent pathways that these different risk factors relate to breast cancer could lead to the development of improved risk assessment tools and prevention strategies.

## Additional files

Additional file 1: Table S1. Pair-wise Pearson correlations between the quantitative breast density measures considered in this work.
Additional file 2: Table S2. Race-stratified, multivariable regression model between absolute area density and the SNP panel, age, BMI and Gail lifetime risk.
Additional file 3: Table S3. Race-stratified, multivariable regression model between area percent density and the SNP panel, age, BMI and Gail lifetime risk.
Additional file 4: Table S4. Race-stratified, multivariable regression model between absolute dense tissue volume and the SNP panel, age, BMI and Gail lifetime risk.
Additional file 5: Table S5. Race-stratified, multivariable regression model between volume percent density and the SNP panel, age, BMI and Gail lifetime risk.
Additional file 6: Table S6. Regression analysis between number of SNP risk alleles and absolute and relative percent area density measures in Caucasian and African-American women after adjusting for age, BMI and Gail lifetime risk for those women with known BMI (i.e., complete data analysis).
Additional file 7: Table S7. Regression analysis between number of SNP risk alleles and absolute and relative percent volume density measures in Caucasian and African-American women after adjusting for age, BMI and Gail lifetime risk for those women with known BMI (i.e., complete data analysis).

## Abbreviations

BI-RADS: Breast imaging-reporting and data system
BMI: Body mass index
FFDM: Full field digital mammography
HIPAA: Health insurance portability and accountability act
IRB: Institutional review board
PD%: Area percent density
SNP: Single nucleotide polymorphism
VD%: Volume percent density
